# Assessing the learning curve of robot-assisted total mesorectal excision: a multicenter study considering procedural safety, pathological safety, and efficiency

**DOI:** 10.1007/s00384-022-04303-7

**Published:** 2023-01-11

**Authors:** T. A. Burghgraef, D. J. Sikkenk, R. M. P. H. Crolla, M. Fahim, J. Melenhorst, M. El Moumni, G. van der Schelling, A. B. Smits, L. P. S. Stassen, P. M. Verheijen, E. C. J. Consten

**Affiliations:** 1 Department of Surgery, University Medical Centre Groningen, Groningen, The Netherlands; 2 Department of Surgery, Meander Medical Centre, Amersfoort, The Netherlands; 3 Department of Surgery, Amphia Hospital, Breda, The Netherlands; 4 Department of Surgery, St Antonius Hospital, Nieuwegein, The Netherlands; 5 Department of Surgery, Maastricht University Medical Centre, Maastricht, The Netherlands

**Keywords:** Robot-assisted surgery, Rectal cancer, Total mesorectal excision, Learning curve

## Abstract

**Purpose:**

Evidence regarding the learning curve of robot-assisted total mesorectal excision is scarce and of low quality. Case-mix is mostly not taken into account, and learning curves are based on operative time, while preferably clinical outcomes and literature-based limits should be used. Therefore, this study aims to assess the learning curve of robot-assisted total mesorectal excision.

**Methods:**

A retrospective study was performed in four Dutch centers. The primary aim was to assess the safety of the individual and institutional learning curves using a RA-CUSUM analysis based on intraoperative complications, major postoperative complications, and compound pathological outcome (positive circumferential margin or incomplete TME specimen). The learning curve for efficiency was assessed using a LC-CUSUM analysis for operative time. Outcomes of patients before and after the learning curve were compared.

**Results:**

In this study, seven participating surgeons performed robot-assisted total mesorectal excisions in 531 patients. Learning curves for intraoperative complications, postoperative complications, and compound pathological outcome did not exceed predefined literature-based limits. The LC-CUSUM for operative time showed lengths of the learning curve ranging from 12 to 35 cases. Intraoperative, postoperative, and pathological outcomes did not differ between patients operated during and after the learning curve.

**Conclusion:**

The learning curve of robot-assisted total mesorectal excision based on intraoperative complications, postoperative complications, and compound pathological outcome did not exceed predefined limits and is therefore suggested to be safe. Using operative time as a surrogate for efficiency, the learning curve is estimated to be between 12 and 35 procedures.

**Supplementary Information:**

The online version contains supplementary material available at 10.1007/s00384-022-04303-7.

## Introduction

Surgical resection is the cornerstone of rectal cancer treatment. This is performed according to the total mesorectal excision (TME) principle, using minimally invasive approaches such as laparoscopic (L-TME), robot-assisted (R-TME) or transanal (TaTME) [1]. Multiple papers have compared L-TME with R-TME and TaTME [2]. If performed by experienced surgeons, R-TME and TaTME may result in an increased primary anastomosis rate, although no difference in postoperative or oncological outcome has been found [3–6].

However, outcomes of minimally invasive surgery are suggested to be influenced by the learning curve [5]. Current literature regarding learning curves of L-TME, R-TME, and TaTME suggests that the length of the learning curve of R-TME is comparable with TaTME and L-TME [7–11]. However, increased intraoperative complication rates and local recurrence rates were seen during the learning curve of TaTME in the Netherlands and Norway [12–14]. This has led to an increase in studies evaluating the learning curve of TaTME and R-TME.

Despite the increased number of papers published on this topic, studies are mostly of poor quality. Series are small and use different definitions for rectal carcinoma, or even include rectal resections for benign diseases as well. Additionally, different outcomes and statistical methods are used to assess the learning curve [15–22]. Mostly, solely operating time is used for assessing the learning curve, without considering clinical outcomes, while the latter are said to be a better surrogate for the learning curve [23]. Additionally, if an appropriate statistical analysis is used, adjusting for case-mix is mostly not done, and length of the learning curve is based on averages of the series (hence, intersurgeons’ differences were measured) instead of using literature-based limits. Therefore, this study aimed to assess the learning curve of R-TME using clinical outcomes primarily, by means of RA-CUSUM analyses using literature-based limits.

## Methods

This is a retrospective multicenter cohort to assess the learning curve of R-TME in four large Dutch teaching hospitals. Learning curves will be assessed for individual surgeons and institutions. A protocol regarding the data-analysis was composed prior to initiation of the study. The manuscript was written according to the STROBE guidelines [24].

### Design

This study involves four robot-assisted centres using the da Vinci system (Intuitive Systems, Sunnyvale, CA, USA). None of the surgeons had prior experience with robot-assisted surgery, and start of the R-TME was preceded by electronic training, animal training, and proctoring of the first 5 procedures led by Intuitive. All surgeons had profound experience with more than 200 L-TMEs and more than 100 open TME procedures performed per surgeon. Centers started with the technique between 2011 and 2016. In the center, the A cases were operated using the DaVinci Xi, performed by one dedicated surgeon. Center B and C used the DaVinci Si, performed by two dedicated surgeons and a dedicated team of OR nurses per center. Center D used the DaVinci Xi, performed by two dedicated surgeons.

### Patients

All consecutive patients that underwent R-TME since its introduction in the specific center were included if they met the following criteria: (1) in need of TME, (2) diagnosed with rectal cancer according to the Sigmoid take-off definition [25], (3) were 18 years or older, (4) were operated in an elective setting with (5) curative intent, and (6) if the performing surgeon had performed > 20 cases during the inclusion period. There were no predefined exclusion criteria. All preoperative decisions and postoperative follow-up were in accordance with the Dutch guideline colorectal carcinoma [26].

### Outcomes

Our primary outcome was to assess the learning curve of the individual surgeons using clinical outcomes. The used clinical outcomes were as follows: intraoperative complications, postoperative major morbidity, and compound pathological outcome. Intraoperative complications were defined as injury to the bladder, ureter, urethra, or vagina, bleeding needing transfusion, serosal injury of the intestine, or any other complication needing intraoperative intervention. Postoperative major morbidity was defined as a postoperative complication within 30 days classified as Clavien–Dindo grade three or higher [27]. The compound pathological outcome was defined as either an irradical resection, which was defined as a circumferential resection margin ≤ 1 mm, or an incomplete TME specimen according to Quirke [28].

Our secondary outcome was estimating the learning curve regarding efficiency, and comparison of outcomes between patients who underwent surgery during the learning curve and after the learning curve had been achieved. For efficiency, operating time was used, which was defined as time from incision until closure. Learning curves were assessed for both individual surgeons as well as institutions.

### Data capturing

Data was captured using the prospective DCRA database of the particular hospital, and missing or unregistered data was added from the local electronic medical record (EMR) system. Data was pseudonymized and registered in the GCP-proof data management system Castor (Ciwit B.V., Amsterdam, the Netherlands). This study was approved by the regional medical ethics review committee (MEC-U, registration number: W19.096), and the local hospitals’ medical ethics review committees.

Patient characteristics that were captured from either the EMR or from the DCRA database included the following: age, sex, body mass index (BMI), ASA (American society of Anesthesiologist) classification, history of abdominal surgery, and history of pelvic surgery. Tumor characteristics included distance of the tumour from the anorectal junction (ARJ) on MRI in centimetres, a low rectal tumour classified according to the LOREC criteria [29], clinical TNM classification, neo-adjuvant (chemo) radiation therapy, and mesorectal fascia (MRF) involvement at preoperative MRI, defined as ≤ 1 mm distance from the MRF. Intraoperative characteristics included surgeon performing the procedure, operating time, type of TME resection (abdominoperineal resection (APR), low anterior resection (LAR) with anastomosis or LAR with an end colostomy), the construction of a stoma, and conversion. Postoperative characteristics included 30-day surgical complications, 30-day mortality, length of stay, readmission, reintervention, wound infection, intra-abdominal abscess, ileus, bleeding, and anastomotic leakage according to the International Study Group of Rectal Cancer [30]. Major morbidity was defined as Clavien–Dindo class three or higher. Additionally, positive circumferential margin, defined as a margin ≤ 1 mm, TME specimen quality according to Quirke and compound pathological outcome was registered [28]. The latter was defined as either a positive circumferential margin or an incomplete TME.

### Statistical analysis

Outcomes were compared between groups using independent sample *T*-test or the Wilcoxon rank sum test for continuous variables, depending on the distribution. For categorical and binary variables, the *X*^2^ test was used. The learning curve was assessed using the risk-adjusted cumulative sum analysis (RA-CUSUM) for intraoperative complications, postoperative major morbidity, and the compound pathological outcome. For operating time, a learning curve cumulative sum analysis (LC-CUSUM) was used to assess the learning curve. A ‘normal’ (RA-)CUSUM assumes an ‘in-control’ state, and signals when the surgeon is out of control, whereas the LC-CUSUM assumes an ‘out-of-control’ state, and signals when the surgeon is in control. In addition, by using the risk-adjusted version, a poor outcome in a high-risk patient will be penalized differently compared to a poor outcome in a low-risk patient [31, 32]. As all surgeons had prior experience with L-TME, and R-TME does not differ significantly with respect to surgical approach (i.e., both use a top-down approach), we used the RA-CUSUM for clinical outcomes. As we assumed that surgeons were not yet in control regarding efficiency, we used the LC-CUSUM for operating time.

To plot the RA-CUSUM, a score was attributed to each surgical procedure: negative when associated with success, and positive when associated with failure. This score was adjusted for patients’ risk factors. When the score equals or exceeds the proficiency limit *h*, the null hypothesis was rejected and the process reached an ‘out-of-control’ state, indicating significantly worse outcome. Additionally, the inverse was plotted as well, with a barrier set at the negative limit h. Additionally, a barrier at 0 was taken into account, preventing the score from being negative, thereby incorporating the assumption of an ‘in-control’ state in which out-of-control performances cannot be compensated with prior results. Limit h was set as a specific percentage of the outcome for the RA-CUSUM. For intraoperative complications the limit was set on a rate of 5.0%, based on previous research showing intra-operative complications between 3 and 5% [3, 33]. For postoperative major morbidity, this was set at 15.0%, based on previous research showing major morbidity (Clavien–Dindo class 3 or higher) between 15 and 20% [3, 34, 35]. For the compound pathological outcome, this was set at 10%, with recent trials showing both incomplete TME as well as positive CRM rate between 3 and 10% [2, 6, 36].

For the LC-CUSUM, the score attributed to each procedure was positive when associated with success, and negative when associated with failure, in contrast to the RA-CUSUM. If the score reached the predefined limit, this would raise a signal indicating ‘in-control’ state. Finally, for the LC CUSUM, limit *h* was set at an operating time of less than 200 min in more than 80% of the cases, based on recently reported outcomes of Dutch R-TME studies [3, 34].

After univariate comparison of outcomes during and after the learning curve, a multivariable logistic regression model with backward selection was used to control for confounding variables for intraoperative morbidity, major postoperative morbidity, and compound pathological outcome. Finally, a multivariable linear regression model with backward selection was used to control for operative time. All analyses were conducted in R using the packages ‘mice’,‘cusum,’ and ‘ggplot2.’

## Results

**Fig. 1:**
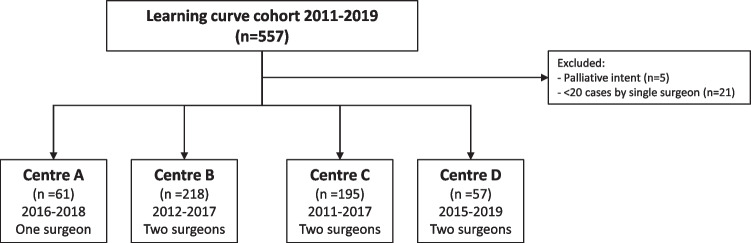
Flow diagram of included patients

In the four participating hospitals, 557 patients were deemed eligible. After exclusion of five patients that were treated with palliative intent, and 21 patients that were treated by two surgeons that operated less than twenty patients during the periods of inclusion, 531 patients remained (Fig. 1).

### Baseline

Significant differences existed regarding the proportion of patients having a history of abdominal surgery, ranging from 18.5 to 43.3% (*p* = 0.02); preoperative mesorectal fascia involvement, ranging from 9.1 to 48.6% (*p* < 0.001); cT4 stage, ranging from 1.0 to 26.7% (*p* < 0.001); and cM1 stage, ranging from 1.9 to 16.7% (*p* < 0.001). Both surgeons in centers C and D showed high rates of cT4 with accordingly high rates of neo-adjuvant therapy. Additionally, center D showed high rates of cM1 tumors (Table 1).

**Table 1:** Baseline characteristics, A1, B1, B2, C1, C2, D1, and D2 represent surgeons from respectively center A, B, C, and D

	Surgeon	Total	A1	B1	B2	C1	C2	D1	D2	*p*
		533	61	119	99	142	53	30	27	
Age (median (IQR))		67 [59, 74]	65 [58, 72]	69 [59, 75]	68 [61, 74]	69 [61, 75]	67 [58, 73]	64 [59, 70]	69 [61, 73]	0.40
BMI (median (IQR))		25 [23, 28]	25 [22, 28]	26 [23, 29]	25 [24, 28]	25 [24, 28]	25 [22, 27]	26 [23, 30]	26 [23, 29]	0.76
Sex (*n*, %)	Female	333 (62.7)	36 (59.0)	78 (65.5)	60 (60.6)	90 (63.4)	35 (66.0)	16 (53.3)	18 (66.7)	0.86
	Male	198 (37.3)	25 (41.0)	41 (34.5)	39 (39.4)	52 (36.6)	18 (34.0)	14 (46.7)	9 (33.3)	
ASA (*n*, %)	1	89 (16.8)	10 (16.4)	16 (13.4)	20 (20.2)	26 (18.3)	14 (26.4)	3 (10.0)	0 (0.0)	0.06
	2	334 (62.9)	39 (63.9)	70 (58.8)	62 (62.6)	94 (66.2)	32 (60.4)	19 (63.3)	18 (66.7)	
	3	108 (20.3)	12 (19.7)	33 (27.7)	17 (17.2)	22 (15.5)	7 (13.2)	8 (26.7)	9 (33.3)	
	4	0 (0.0)	0 (0.0)	0 (0.0)	0 (0.0)	0 (0.0)	0 (0.0)	0 (0.0)	0 (0.0)	
History of abdominal surgery (*n*, %)		160 (30.1)	25 (41.0)	22 (18.5)	29 (29.3)	48 (33.8)	15 (28.3)	13 (43.3)	8 (29.6)	0.02
History of pelvic surgery (*n*, %)		48 (9.0)	9 (14.8)	9 (7.6)	10 (10.1)	13 (9.2)	1 (1.9)	4 (13.3)	2 (7.4)	0.33
Distance tumor on MRI (median (IQR))		5 [3, 8]	4.00 [2, 7]	5 [3, 8]	5 [3, 8]	6 [3, 9]	6 [4, 8]	4 [2, 7]	4 [2, 7]	0.13
MRF involvement (%)	Yes	160 (30.1)	11 (18.0)	31 (26.1)	9 (9.1)	69 (48.6)	18 (34.0)	14 (46.7)	8 (29.6)	< 0.001
	Missing	37 (7.0)	2 (3.3)	14 (11.8)	12 (12.1)	6 (4.2)	3 (5.7)	0 (0.0)	0 (0.0)	
Low rectal tumor (*n*, %)	Yes	297 (55.9)	37 (60.7)	77 (64.7)	59 (59.6)	59 (41.5)	29 (54.7)	18 (60.0)	18 (66.7)	0.005
	Missing	25 (4.7)	7 (11.5)	4 (3.4)	4 (4.0)	8 (5.6)	1 (1.9)	1 (3.3)	0 (0.0)	
cT (*n*, %)	1	14 (2.6)	6 (9.8)	1 (0.8)	3 (3.0)	3 (2.1)	1 (1.9)	0 (0.0)	0 (0.0)	< 0.001
	2	144 (27.1)	20 (32.8)	40 (33.6)	47 (47.5)	17 (12.0)	8 (15.1)	4 (13.3)	8 (29.6)	
	3	290 (54.6)	33 (54.1)	63 (52.9)	43 (43.4)	84 (59.2)	32 (60.4)	18 (60.0)	17 (63.0)	
	4	71 (13.4)	2 (3.3)	11 (9.2)	1 (1.0)	36 (25.4)	11 (20.8)	8 (26.7)	2 (7.4)	
	Missing	12 (2.3)	0 (0.0)	4 (3.4)	5 (5.1)	2 (1.4)	1 (1.9)	0 (0.0)	0 (0.0)	
cN (*n*, %)	0	217 (40.9)	34 (55.7)	48 (40.3)	49 (49.5)	52 (36.6)	14 (26.4)	8 (26.7)	12 (44.4)	< 0.001
	1	167 (31.5)	20 (32.8)	40 (33.6)	31 (31.3)	45 (31.7)	22 (41.5)	6 (20.0)	3 (11.1)	
	2	137 (25.8)	7 (11.5)	26 (21.8)	16 (16.2)	43 (30.3)	17 (32.1)	16 (53.3)	12 (44.4)	
	Missing	10 (1.9)	0 (0.0)	5 (4.2)	3 (3.0)	2 (1.4)	0 (0.0)	0 (0.0)	0 (0.0)	
cM (*n*, %)	0	484 (91.1)	52 (85.2)	112 (94.1)	97 (98.0)	126 (88.7)	52 (98.1)	23 (76.7)	22 (81.5)	< 0.001
	1	33 (6.2)	8 (13.1)	7 (5.9)	2 (2.0)	6 (4.2)	1 (1.9)	5 (16.7)	4 (14.8)	
	Missing	14 (2.6)	1 (1.6)	0 (0.0)	0 (0.0)	10 (7.0)	0 (0.0)	2 (6.7)	1 (3.7)	
Neo-adjuvant therapy (*n*, %)	None	155 (29.2)	30 (49.2)	32 (26.9)	41 (41.4)	26 (18.3)	10 (18.9)	7 (23.3)	9 (33.3)	< 0.001
	Radiotherapy	194 (36.5)	19 (31.1)	58 (48.7)	35 (35.4)	51 (35.9)	17 (32.1)	7 (23.3)	7 (25.9)	
	Chemoradiation	182 (34.3)	12 (19.7)	29 (24.4)	23 (23.2)	65 (45.8)	26 (49.1)	16 (53.3)	11 (40.7)	

*IQR *interquartile range, *BMI *body mass index, *ASA *American Society of Anesthesiologist Classification, *MRI *magnetic resonance imaging, *MRF *mesorectal fascia, *cT *clinical tumor stage, *cN *clinical nodal stage, *cM *clinical metastasis stage, *APR *abdominoperineal resection, *LAR *low anterior resection

### Learning curve assessment

**Fig. 2:**
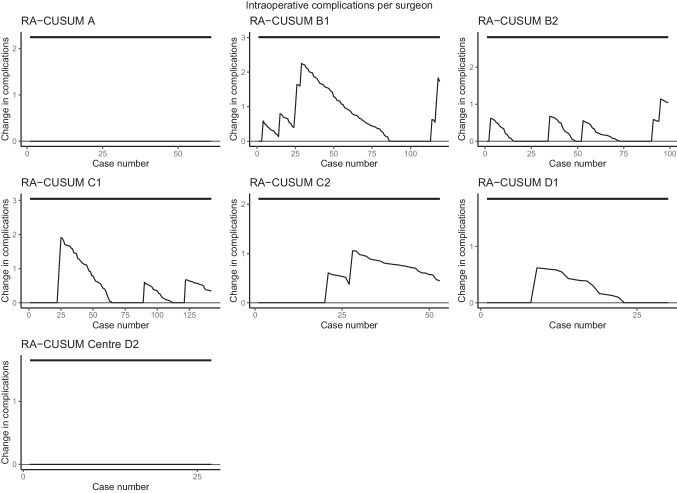
Risk-adjusted CUSUM based on intraoperative complications for individual surgeons of centers A, B, C, and D. The upper limit is set at 5%, based on literature-reported intraoperative complication rates, and detects a significant increase in complications

**Fig. 3:**
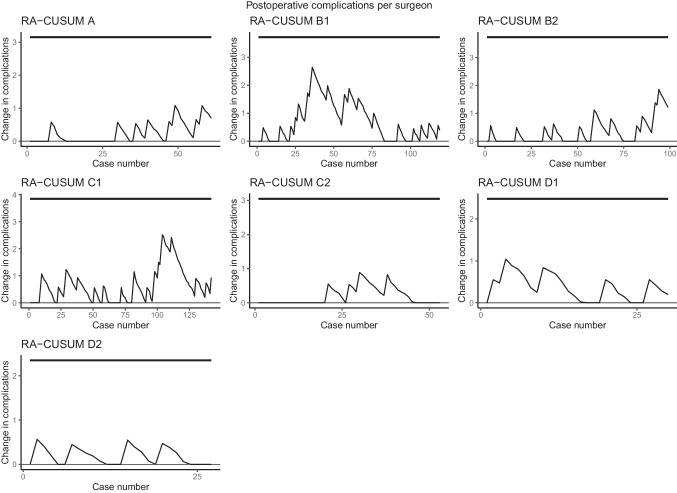
Risk-adjusted CUSUM based on major postoperative complications for individual surgeons of centers A, B, C, and D. The upper limit is set at 15%, based on literature-based postoperative complication rates, and detects a significant increase in complications

**Fig. 4:**
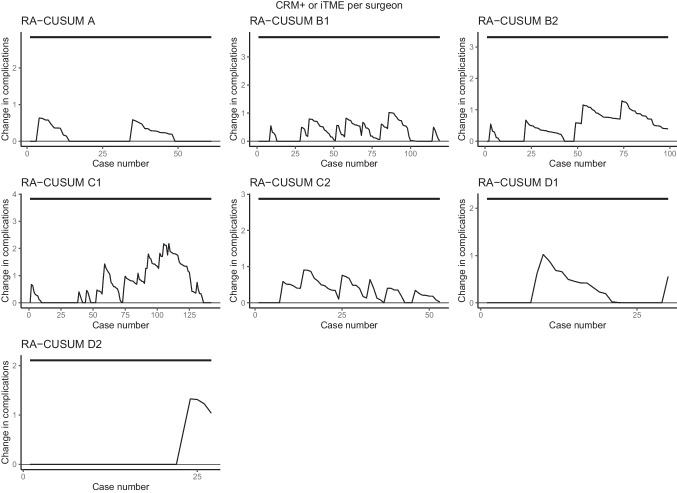
Risk-adjusted CUSUM based on compound pathological outcome for individual surgeons of centers A, B, C, and D. The upper limit is set at 10%, based on literature-based rates of incomplete TME and positive CRM rates, and detects a significant increase in compound pathological outcome. iTME: incomplete total mesorectal excisio; CRM + : positive circumferential margin

**Fig. 5:**
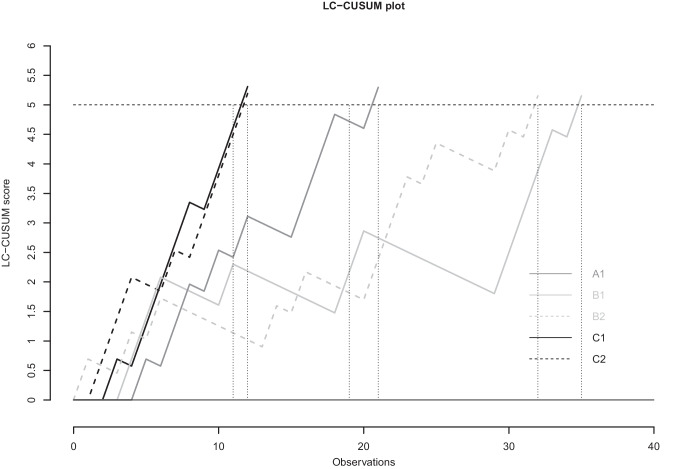
Learning curve (LC)- CUSUM based on operative time for individual surgeons of centers A, B, C, and D. The upper limit was set so that it signaled if more than 80% of the procedures was performed within 200 min

With regard to intraoperative complications, major postoperative complications, and compound pathological outcome, all seven surgeons stayed within pre-specified limits of the ‘in-control’ state. (Figs. 2, 3, and 4). When only taking into account the center, all centers stayed within the limits as well (Supplemental Figs. 1, 2, and 3). The LC-CUSUM for operative time was only performed in centers A, B, and C, as operating time was missing in up to 50% of the cases in center D. Limit *h*, indicating an ‘in-control’ state, was reached at 12, 12, 21, 32, and 35 procedures for surgeon C1, C2, A1, B2, and B1, respectively (Fig. 5).

### Before and after comparison

Patients who underwent surgery before the learning curve was reached and had received significantly more neo-adjuvant therapy compared to patients after the learning curve (80.4% versus 67.7%, *p* = 0.03) (Supplemental Table 1). Regarding outcomes during and after the learning curve, operating time was significantly longer in the group of patients operated during the learning curve (221 min [189, 253] versus 178 min [139, 225], *p* < 0.001). Except for overall surgical complications, clinical outcomes were comparable between the two groups. A significantly higher rate of postoperative surgical complications was observed in the group of patients who underwent R-TME after the learning curve was achieved (21.4% vs. 36.5%, *p* = 0.004). However, major morbidity was not significantly different (9.8% versus 15.3%, *p* = 0.19). These results were confirmed in a multivariable logistic regression model: Patients operated after the learning curve was achieved were independently associated with a higher overall 30-day surgical morbidity (OR: 2.39 [95% CI: 1.43–4.13]), while major morbidity, intraoperative morbidity, and compound pathological outcome were comparable (Table 2).

**Table 2:** Outcomes of patients operated by surgeons during and after achieving the learning curve

		During learning curve	After learning curve	*p*
		112	362	
Operating time (median (IQR))		221 [189, 253]	178 [139, 225]	< 0.001
Conversion (*n*, %)		4 (3.6)	14 (3.8)	0.54
Intra-operative complications (*n*, %)	Ureter/urethra injury (*n*, %)	1 (0.9)	2 (0.6)	0.77
	Bladder injury (*n*, %)	0 (0.0)	2 (0.6)	
	Vagina injury (*n*, %)	2 (1.8)	2 (0.6)	
	Other	4 (3.6)	8 (2.2)	
Surgical procedure (%)	APR	45 (40.2)	125 (34.5)	0.24
	LAR + anastomosis	56 (50.0)	212 (58.6)	
	LAR + stoma	11 (9.8)	25 (6.9)	
30-day surgical morbidity (*n*, %)		24 (21.4)	132 (36.5)	0.004
	Anastomotic leakage (*n*, %)	3 (5.3)	28 (13.1)	0.15
	Abcess (*n*, %)	2 (1.8)	23 (6.4)	0.10
	Bleeding (*n*, %)	2 (1.8)	14 (3.9)	0.44
	Ileus (*n*, %)	12 (10.7)	66 (18.2)	0.08
	Wound infection (*n*, %)	4 (3.6)	18 (5.0)	0.72
Clavien–Dindo > IIIa (*n*, %)		11 (9.8)	55 (15.3)	0.19
30-day mortality (*n*, %)		0 (0.0)	7 (1.9)	0.30
LOS (median (IQR))		6 [5, 8]	6 [4, 11]	0.71
Reintervention (*n*, %)		10 (8.9)	55 (15.2)	0.13
Readmission (*n*, %)		15 (13.4)	64 (17.7)	0.36
CRM + (*n*, %)		7 (6.2)	20 (5.5)	0.96
incomplete TME (*n*, %)		1 (0.9)	12 (3.3)	0.30
Compound pathological outcome (*n*, %)		8 (7.1)	29 (8.0)	0.92

*IQR *interquartile range, *LOS *length of (hospital) stay, *CRM* + positive circumferential margin, *TME *total mesorectal excision

## Discussion

The aim of this study was to evaluate the learning curve of R-TME, using literature-based limits, with risk-adjusted analyses. The learning curve was primarily assessed using clinical outcomes, while secondly operating time was used. All surgeons and centers stayed within the prespecified literature-based limits of an ‘in-control’ state for intraoperative complications, major postoperative complications, and compound pathological outcomes. In addition, an ‘in-control’ state for operating time was reached after 12–35 procedures. Moreover, no differences regarding intraoperative complications, major postoperative complications, and compound pathological outcome were observed between patients operated during and after the learning curve, which was based on operative time (Table 2).

Both for individual surgeons and for the institutions, the outcomes suggest that a learning curve for clinical outcomes, estimating safety, could not be distinguished. This is in contrast to former studies showing longer learning curves [7, 10, 11, 16, 34, 37–39]. This could be explained by several reasons. First, surgeons participating in this cohort study were experienced surgeons regarding open and laparoscopic TME, which might have resulted in the absence of a distinguishable learning curve. However, most surgeons starting with R-TME have experience with either open or L-TME; therefore, this study reflects clinical practice. Second, previous studies mostly define the length of the learning curve solely on operative time [37, 38]. However, this is a rather poor outcome for assessing the learning curve [23, 40]. Second, studies that do use clinical outcomes to assess the learning curve, mostly use CUSUM or RA-CUSUM analyses, which is an adequate analysis. These analyses are primarily used to detect a deviation from an ‘in-control’ state to either a significant decrease or a significant increase in the occurrence of an event compared to literature based limits [31, 40, 41]. But, most studies evaluating length of the learning curve with (RA)-CUSUM analyses use deflection of the learning curve to assess the length, instead of the actual goal of the analysis: checking whether there is a significant deviation from an ‘in-control’ state. Using the deflection of the learning curve for assessing the length of the learning curve may be false, as the outcome may still remain within limits [10, 11, 39]. Thereby, the actual analysis is performed well, but it is mostly misinterpreted. Additionally, it is suggested that increased length of the specific series of the surgeon or center is associated with increased length of the learning curve if deflection of the learning curve is used, rather than predefined limits [23, 31, 39]. Therefore, these results might not actually reflect length of the learning curve.

The only three studies assessing the learning curve that use (RA)-CUSUM analyses for clinical outcomes with literature-based limits, involve studies evaluating TaTME surgery [8, 9, 12]. These studies suggested an ‘out-of-control’ state from initiation until the 25^th^–55^th^ cases, based on intraoperative and postoperative complications [8, 9, 12]. All three studies assessing the learning curve of TaTME used a (RA)-CUSUM analysis. However, as the bottom-up approach used in TaTME is new to most surgeons, preferably a LC-CUSUM analysis should be used as this analysis assumes an ‘out-of-control’ state. Despite the low number of studies, these results suggest that the learning curve of TaTME comes with additional intraoperative and postoperative morbidity.

As clinical outcomes stayed within predefined, literature-based limits in this study, length of the learning curve could not be based on clinical outcomes. Therefore, we used operative time, as a secondary outcome, to assess length of the learning curve. The LC-CUSUM analysis showed length of the learning curve to range between 12 and 35 cases for individual surgeons. Previous studies assessing the learning curve based on operative time, using a limit are scarce. A R-TME study suggested a length of 19 cases, while a TaTME study suggested a length of 39 cases [8, 42]. Outcomes of studies assessing length of the learning curve based on operative time, using deflection of the curve, vary widely between 8 and 110 cases [43]. The wide variety is due to not taking into account patient characteristics or surgeon experience, while deflection of the curve is probably not the appropriate way of defining length of the learning curve [44].

When comparing patients operated during the learning curve with patients operated after the learning curve, we saw a significant decrease in operative time. Several studies that based the learning curve on operative time, showed a significant decrease in operative time [10, 11, 38]. This unsurprising finding could be explained by the fact that length of the learning curve was defined by operative time, implicating a significant difference regarding this outcome. In line with other studies and the RA-CUSUM analysis, major postoperative complications, intraoperative complications, and compound pathological outcome did not differ during and after the learning curve of R-TME [10, 11, 16, 39, 45]. Despite the fact that no difference in major postoperative complications was observed, we did observe a significant difference with respect to overall 30-day surgical morbidity. In fact, overall surgical morbidity was higher in the group of patients who underwent surgery after the learning curve had been achieved, even after correcting for confounding factors. Perhaps residual confounding was present, thereby case-mix could have resulted in more difficult cases after the learning curve had been achieved, with a consequent higher proportion of surgical complications. Another explanation might be the fact that this is a retrospective study, with a significant risk of reporting bias, especially for minor complications, as these are mostly less well documented, while major morbidity is generally well documented, as the latter is defined as treated with surgical or radiological interventions, which are generally well documented.

To our knowledge this is the first study assessing the individual learning curves of R-TME surgeons by means of RA-CUSUM analyses of clinical variables, using literature-based limits. Although this study suggests that the learning curve of R-TME, for surgeons experienced in open or L-TME, is not associated with additional morbidity, and length of the learning curve for operating time is between 12 and 35 procedures, certain limitations should be taken into account. First, it should be appreciated that the learning ability and experience of the surgeons varies widely between surgeons, while statistical analyses cannot control for these variables. Therefore, these analyses reflect a theoretical simplification of a complex process. Second, this retrospective cohort analysis comes with reporting bias and confounding. We tried to control for confounding factors by using RA-CUSUM analyses for assessing the learning curve and a multivariable regression analysis when comparing outcomes during and after the learning curve. However, residual confounding might be present. Third, although most of the series were of considerable length, two surgeons only performed 30 and 27 procedures while operating times were missing in more than 50% of these cases. Therefore, we excluded these two surgeons regarding the LC-CUSUM analysis. Fourth, although we used literature-based limits for the RA-CUSUM and LC-CUSUM, especially the limit for operating time might be debatable. Not only case-mix influences this, as this is a variable reflecting efficiency which is affected by more than only the surgeon performing the procedure. In addition, cultural, logistic, and financial factors may differ between centers and could influence these outcomes as an effect. Fifth, a significantly higher rate of patients operated before the learning curve were treated with neo-adjuvant therapy, due to change of the Dutch Guideline in 2014 [46]. This might have influenced outcomes. Finally, the surgeons participating in this cohort study were surgeons with experience in L-TME and open TME, which might have caused the absence of a learning curve with regard to clinical outcomes.

Concluding, this is the first study assessing the learning curve of R-TME by means of a RA-CUSUM analysis with literature-based limits, using clinical variables. This study suggests that the learning curve of R-TME is safe in terms of intraoperative complications, major postoperative complications and pathological outcomes. The learning curve based on efficiency might range between 12 and 35 procedures for R-TME.

### Supplementary Information

Below is the link to the electronic supplementary material.

Supplementary file1 (DOCX 31 KB)

Supplementary file2 (DOCX 32 KB)

Supplementary file3 (DOCX 31 KB)

Supplementary file4 (DOCX 20 KB)

## Data Availability

Data is available upon reasonable request.
