# Supplementary material for: Assessing the learning curve of robot-assisted total mesorectal excision: a multicenter study considering procedural safety, pathological safety, and efficiency
Source: Int J Colorectal Dis. 2023 Jan 11;38(1):9. doi: 10.1007/s00384-022-04303-7 (PMC9834356; doi:10.1007/s00384-022-04303-7)
Supplement: Supplementary file 1 — Supplementary file1 (DOCX 31 KB) [file 384_2022_4303_MOESM1_ESM.docx]

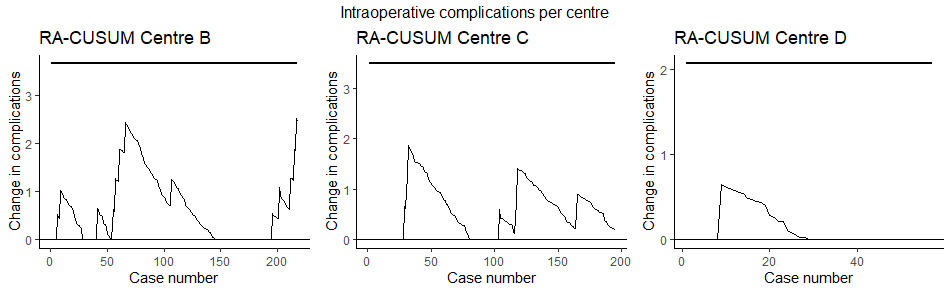


**Supplemental figure 1:** Risk-adjusted CUSUM for centers B, C and D. Upper and lower limits are based on literature-based intraoperative complication rates. Upper limits detect a significant increase in complications, while the lower limits detect a significant decrease in intraoperative complications
