# Supplementary material for: Assessing the learning curve of robot-assisted total mesorectal excision: a multicenter study considering procedural safety, pathological safety, and efficiency
Source: Int J Colorectal Dis. 2023 Jan 11;38(1):9. doi: 10.1007/s00384-022-04303-7 (PMC9834356; doi:10.1007/s00384-022-04303-7)
Supplement: Supplementary file 4 — Supplementary file4 (DOCX 20 KB) [file 384_2022_4303_MOESM4_ESM.docx]

|  |  | During learning curve | After learning curve | P |
| --- | --- | --- | --- | --- |
|  |  | 112 | 362 |  |
| Age (median [IQR]) |  | 65 [59, 73] | 68 [60, 75] | 0.22 |
| BMI (median [IQR]) |  | 25 [23, 29] | 26 [23, 28] | 0.52 |
| Sex (n, %) | Male | 68 (60.7) | 231 (63.8) | 0.63 |
|  | Female | 44 (39.3) | 131 (36.2) |  |
| ASA (n, %) | 1 | 20 (17.9) | 66 (18.2) | 0.99 |
|  | 2 | 70 (62.5) | 227 (62.7) |  |
|  | 3 | 22 (19.6) | 69 (19.1) |  |
|  | 4 | 0 (0.0) | 0 (0.0) |  |
| History of abdominal surgery (n, %) |  | 36 (32.1) | 103 (28.5) | 0.52 |
| History of pelvic surgery (n, %) |  | 13 (11.6) | 29 ( 8.0) | 0.33 |
|  |  |  |  |  |
| Distance tumor on MRI (median [IQR]) |  | 5 [2, 8] | 6 [3, 8] | 0.22 |
| MRF involvement (%) | Yes | 63 (61.2) | 198 (57.1) | 0.53 |
|  | Missing | 9 (8.0) | 15 (4.1) |  |
| Low rectal tumor (n, %) | Yes | 25 (28.1) | 113 (32.5) | 0.51 |
|  | Missing | 23 (20.5) | 14 (3.9) |  |
|  |  |  |  |  |
| cT (n, %) | 1 | 5 ( 4.9) | 9 ( 2.5) | 0.06 |
|  | 2 | 26 (25.2) | 106 (29.5) |  |
|  | 3 | 65 (63.1) | 190 (52.9) |  |
|  | 4 | 7 ( 6.8) | 54 (15.0) |  |
|  | Missing | 9 (8.0) | 3 (0.8) |  |
| cN (n, %) | 0 | 47 (45.2) | 150 (41.7) | 0.80 |
|  | 1 | 33 (31.7) | 125 (34.7) |  |
|  | 2 | 24 (23.1) | 85 (23.6) |  |
|  | Missing | 8 (7.1) | 2 (0.6) |  |
| cM (n, %) | 0 | 105 (95.5) | 334 (94.6) | 0.92 |
|  | 1 | 5 ( 4.5) | 19 ( 5.4) |  |
|  | Missing | 2 (1.8) | 9 (2.5) |  |
|  |  |  |  |  |
| Neo-adjuvant therapy (n, %) | None | 22 (19.6) | 117 (32.3) | 0.03 |
|  | Radiotherapy | 50 (44.6) | 130 (35.9) |  |
|  | Chemoradiation | 40 (35.7) | 115 (31.8) |  |

**Supplemental table 1**: Baseline characteristics of patients operated by surgeons during and after achieving the learning curve. Surgeon D1 and D2 were not taken into account since the learning curve could not be assessed. IQR: interquartile range, BMI: body mass index, ASA: American society of anesthesiologist classification, MRI: magnetic resonance imaging, MRF: mesorectal fascia, cT: clinical tumor stage, cN: clinical nodal stage, cM: clinical metastasis stage, APR: abdominoperineal resection, LAR: low anterior resection.
